# Supplementary material for: Justifying Euthanasia: A Qualitative Study of Veterinarians’ Ethical Boundary Work of “Good” Killing
Source: Animals (Basel). 2023 Aug 4;13(15):2515. doi: 10.3390/ani13152515 (PMC10416879; doi:10.3390/ani13152515)
Supplement: Supplementary file 1 [file animals-13-02515-s001.zip › animals-2432933-supplementary.pdf]

Interview Guide - Veterinary Killing

[Place] – [Date]

Opening:

- First, I would like you to briefly introduce yourself: Who are you, and where are you currently working?
- I am interested in your professional background: What other career steps have you taken before your current position?

Block I: Specific Work Actions & Killing Practices

- What do you enjoy most about your work?
- Please tell us what you do daily: What is a typical workday like for you?
- Why did you specialize in this field (e.g., farm animal medicine)?
- What attracted you to work with this species (e.g., cattle)?
- In what professional situations do you become involved in killing animals?
- What role does the death or killing of animals play in your work?
- Can you tell me in detail how the killing takes place?
- Could you please show me how the killing is done with your hands? (Please explain in detail what you do, how, and why you do it).
- What case in the last few weeks stands out in your mind? (Can you explain how this case is different from others? / Why?)
- How do you handle the animal in these situations?
  (Is your handling different when the client is present or absent? Is handling the animal different for killing than other treatments?)

Block II: Professional Biography

- Why did you decide to study veterinary medicine? (Motivations, experiences, etc.)
- Do you remember the first time an animal was killed in your presence? Could you please tell me about it?
- Can you remember the first time you killed an animal? Could you please tell me about it?
- Has your attitude toward killing changed over the years?
- Based on your experience, what advice would you give your former self today?
- How have your thoughts and feelings about killing animals changed throughout your professional experience? (How have you learned to deal with it / are there things that make it easier?)

- How did your studies deal with death and killing? How did your studies prepare you for the situation [of killing]?
- How does your social environment react to the fact that you kill animals for a living? [e.g., family, friends, strangers].
- Do you discuss killing animals with others?
  If yes, with whom and how do you discuss this aspect of your work?
  If not, do you need to talk about it?
- When new, young colleagues are brought in, how do you teach them about killing?

Block III: General Veterinary Medicine

- Have you ever looked at guidelines and decision aids for killing? If so, how have they helped you in your work?
  (Have you ever looked at the BTK Code of Ethics? If so, how relevant is it to your work?)
- What do you think is the role of veterinary medicine in society? How do you see the part of veterinary medicine today?
- In your opinion, has the killing of animals (in veterinary medicine) changed during your professional experience?
- Keyword Anthropocene, i.e., the change in the earth's history caused by humans (climate change, mass extinctions, etc.) - What could humans learn from veterinary medicine? What role could veterinary medicine play?
- Think about what we have discussed today: What would you like to see in the future of veterinary medicine? (And about the killing of animals?)

Closing

- Can you think of anything else about the killing of animals in veterinary medicine that you haven't already told us? Or would you like to add something?
- Is there anything else you would like to say that is important to you but has not been mentioned in our interview? Something you were expecting, but I didn't ask.
- How did it feel for you to talk about killing?
- Do you have any other questions for me?
- Can you think of any colleagues: inside who might be interested in talking to me?

Thank you for this interview!
